# Enhancing well‐being and alleviating depressive symptoms in people with HIV/AIDS: An intervention based on if–then plans with self‐affirming cognitions

**DOI:** 10.1111/aphw.12357

**Published:** 2022-03-16

**Authors:** Patryk Łakuta, Dagny Krankowska, Przemysław Marcinkiewicz, Monika Bociąga‐Jasik, Agnieszka Komorska‐Błażewicz

**Affiliations:** ^1^ Institute of Psychology SWPS University of Social Sciences and Humanities Warsaw Poland; ^2^ Institute of Psychology Cardinal Stefan Wyszyński University Warsaw Poland; ^3^ Department of Infectious and Tropical Diseases and Hepatology Medical University of Warsaw, Hospital for Infectious Diseases Warsaw Poland; ^4^ The Infant Jesus Clinical Hospital University Clinical Centre, Medical University of Warsaw Warsaw Poland; ^5^ Department of Infectious Diseases Jagiellonian University Medical College Kraków Poland; ^6^ Department of Infectious Diseases University Hospital in Krakow Kraków Poland

**Keywords:** depression, HIV, implementation intention, mental health, self‐affirmation, well‐being

## Abstract

Effective antiretroviral treatment has increased the life expectancy of people living with HIV, and currently, the challenges of prominent importance appear to be mental health issues. This preregistered study among adults living with HIV/AIDS investigated the effectiveness of a brief self‐affirmation intervention framed in terms of if–then plans (i.e. self‐affirming implementation intentions [S‐AII]) against both active and non‐active control conditions, forming non‐affirming implementation intentions and mere goal intentions, respectively. The primary outcomes were defined as a reduction of depressive symptoms and enhancement of well‐being, along with secondary outcomes as positive other‐ and self‐directed feelings. A total of 162 individuals were assessed for eligibility, and 130 (aged 18–74 years) were randomized to the study conditions. Intervention effects were estimated through intention‐to‐treat analysis, using linear mixed models. The S‐AII intervention yielded improvements in overall well‐being over 2 weeks (*d* = .23), primarily driven by positive changes in emotional (*d* = .24) and social (*d* = .30) dimensions of well‐being. There were no significant differences in depression or secondary outcomes. Based on a minimal clinically important difference index, the S‐AII intervention resulted in improvement in well‐being in approximately 40 percent of participants. Nevertheless, further systematic research is needed to optimize self‐affirmation‐interventions, before their application in real‐life contexts.

## INTRODUCTION

Effective antiretroviral treatment (ART) has increased the life expectancy of people living with HIV, and currently, non‐AIDS‐related comorbidities, including mental health diseases, are the challenges of prominent importance. There is sound evidence that people living with HIV/AIDS (PLWHA) are at increased risk of depression and suicidal ideation (cf. Catalan et al., 2011; Ciesla & Roberts, 2001; Feuillet et al., 2017; Rezaei et al., 2019; Sherr et al., 2011; Tran et al., 2019). In Europe, the prevalence rate of depression in patients with HIV/AIDS is estimated at 22 percent (95% confidence interval [CI]: 17% to 27%) (Rezaei et al., 2019). Of importance, many studies have shown that adult PLWHA with mental health difficulties are at increased risk of HIV transmission and medication nonadherence and are more likely to experience cumulative life course impairment (e.g. Gonzalez et al., 2011; MacNeil et al., 1999; Sweat et al., 2000; Tran et al., 2019). Depression is most strongly related to nonadherence to antiretroviral treatment. Meta‐analytical evidence has suggested that the link is consistent across samples and over time and is not limited to those with major depression, and, importantly, the effect is not inflated by self‐report bias (Gonzalez et al., 2011; see also Tao et al., 2018; Uthman et al., 2014). Therefore, developing psychological interventions aimed at reducing depressive symptoms, even at subclinical levels, should be a current research priority.

The question then arises as to whether psychological theories have anything to offer in terms of optimizing mental health and well‐being and whether it is possible to develop a brief psychological intervention for PLWHA that can truly enhance their well‐being and mental health outcomes. Based on the growing empirical evidence on self‐affirmation interventions, we tested whether encouraging to cultivate a sense of self as worthy, adequate, and efficacious (Cohen & Sherman, 2014; Steele, 1988) can provide improvements on mental health indicators, namely, levels of depression and well‐being. The central aim of the present research was to evaluate the effectiveness of a brief self‐help psychological intervention—self‐affirmation intervention within planning mode, known as self‐affirming implementation intentions (S‐AII), in terms of better mental health outcomes among PLWHA. To date, despite theoretical promise, the effectiveness of such interventions for PLWHA's mental health outcomes has not been examined.

The basic prediction of self‐affirmation theory (Steele, 1988; see also Cohen & Sherman, 2014; Sherman, 2013; Sherman & Cohen, 2006) is that bolstering self in one important domain should buffer the impact of threats in another. Self‐affirming personally important domains makes an individual reflect on such things as personally important values and principles, strengths and attributes, or social relationships, providing a broader perspective on one's self (and also on possible strategies and activities). Critcher and Dunning (2015) suggested that when an individual experiences a threat to an important aspect of self‐conception/image, a threatened identity dominates the working self‐concept, thereby providing a narrow perspective on the self with an accompanying depressed sense of worth. Self‐affirming, as presumed, injects into the working self‐concept compensating sources of self‐evaluation, reminding people that the threatened domain is not all that defines the self. With more alternative identities (e.g. resourceful, honest, and a good partner/worker) active to help break a constricted self and dampen the evaluative impact of the threat, self‐worth may restore with much broader dispositional self‐views (cf. Critcher & Dunning, 2015).

There are a number of techniques for self‐affirmation induction (cf. McQueen & Klein, 2006). In the present study, we employed a standardized self‐affirmation intervention (Armitage et al., 2011; see also Armitage, 2016; Łakuta, 2020, 2021; Morgan & Atkin, 2016; Morgan & Harris, 2015), in which an individual forms an if–then plan with self‐affirming cognitions. This intervention, framed in terms of implementation intentions (Gollwitzer, 2014), provides a brief, simple, and efficient way to self‐affirm. Importantly, within the planning mode via implementation intentions, the procedure enables the creation of strong associative links between the critical situation (*if* part) and the goal‐directed response (*then* part), allowing an individual to automatically initiate the planned response (i.e. self‐affirming) once encountering the critical situation (i.e. self‐threat). The intervention thus employs a self‐affirmation paradigm and also makes use of the if–then structure of implementation intentions (Gollwitzer, 2014). The consequences of forming implementation intentions, primarily in terms of the notion of strategic automaticity, are here the most noteworthy, supported by a vast body of evidence, also from neuroscientific investigations (Martiny‐Huenger et al., 2017; Wieber et al., 2015; for a review, see Bieleke et al., 2021). Forming implementation intentions builds a self‐regulation strategy of great potency that can enhance goal attainment by facilitating the automatic initiation of goal‐directed responses upon encountering critical situations. In this respect, thus, it may facilitate creating a new habit/tendency to spontaneously call to mind self‐affirming cognitions when a stressor arises (cf. Brady et al., 2016; Emanuel et al., 2018). Moreover, the key advantage of using the self‐regulation strategy of forming implementation intentions seems that it could make self‐affirming cognitions readily available, that is, when the self‐system is threatened. The issue of timeliness seems very important. Within the Trigger and Channel Framework, Ferrer and Cohen (2019) suggested that to expect beneficial self‐affirmation effects, besides the presence of threat and the availability of resources (e.g. instrumental content), the intervention should occur near the time the threat emerges.

To date, positive effects of self‐affirmations have been shown for a variety of contexts that are relevant for mental health and well‐being (for a review, see Howell, 2017). Several studies have found that affirming core values upon threat broadens the perceived bases of self‐worth (Critcher & Dunning, 2015), reduces anxiety, helps people to deal with stressful situations (Cohen & Sherman, 2014; Morgan & Atkin, 2016; Morgan & Harris, 2015; Sherman, 2013), and also increases self‐directed (Lindsay & Creswell, 2014) and other‐directed positive feelings, suggesting the mediating mechanisms of self‐affirmation effects on mental well‐being by positive affect (Crocker et al., 2008; Thomaes et al., 2012). However, there still is sparse evidence on such effects in individuals dealing with chronic conditions. This is important because if we are truly interested in whether (and how) self‐affirming can generate both positive effects—a net benefit on psychological functioning (i.e. boosting of well‐being), and a reduction of negative mental states (e.g. depressive symptoms), it would be best to demonstrate and examine its effectiveness in circumstances where people are not exposed to induced or artificial self‐threats. Essentially, such investigations are needed to further support (or refute) self‐affirmation intervention status as a mental health intervention. Although as noted above, self‐affirmation interventions have been shown to yield positive effects across the literature, the presence of null and negative findings have also been reported (e.g. Engeln & Imundo, 2020; Jessop et al., 2018; Łakuta, 2022).

### The present study

The study aimed to address gaps in the literature on the applicability of self‐affirmation theory by testing the intervention in a truly at‐risk population, using an RCT design with both passive and active comparison groups to provide robust evidence for its presumed effectiveness. From a theoretical standpoint, the research aimed to demonstrate that prompting people to self‐affirm may help them attain significant improvement in mental health outcomes—both the decrease of negative outcomes in terms of depressive symptoms and the enhancement of positive outcomes in terms of well‐being. Given that self‐affirmation prompts people to reflect on their values, strengths, and/or social relations and experiences most important to them, it has been suggested that it may also encourage them to engage in activities that are congruent with those values—activities that are happiness‐enhancing (boosting hedonic well‐being) and/or reinforce vital aspects of eudaimonic well‐being as positive relations with others, the fulfillment of psychological needs, and the experience of meaning and purpose in life (for a review, see Howell, 2017). Aligned with this reasoning, we adopted the conceptualization of well‐being as involving both hedonic and eudaimonic aspects (cf. Seligman, 2011; Wong, 2011). Furthermore, in terms of secondary outcomes, to understand more about the way in which self‐affirmation interventions operate, we also evaluated the effects of the intervention on positive self‐ and other‐directed feelings.

To our knowledge, this is the first preregistered study among PLWHA to examine the effectiveness of the intervention based on if–then plans with self‐affirming cognitions (i.e. S‐AII) compared with an active control group employing distraction strategies (non‐affirming implementation intentions [N‐AII]) and a condition in which participants form mere goal intentions (MGI; passive control). Four preregistered hypotheses^1^ were established. Primarily, the S‐AII intervention was hypothesized to be superior relative to both N‐AII and MGI in improving depression (H1) and well‐being (H2) at 2 weeks posttreatment. Turning to the secondary outcomes, as suggested in prior research (Crocker et al., 2008; Lindsay & Creswell, 2014; Thomaes et al., 2012), it was hypothesized that participants in the S‐AII intervention condition would experience significantly higher levels of positive self‐directed feelings (H3) and other‐directed feelings (H4) at 2 weeks post‐intervention.

## METHODS

### Participants and procedure

This randomized, controlled, three‐arm, parallel‐group trial was conducted between 2020 and 2021. The study protocol was preregistered on the Open Science Framework^1^ before any data were collected. Recruitment was carried out in several departments and outpatient clinics treating PLWHA in Poland, as well as through online venues (i.e. social media) in self‐referral communities.^2^ To be eligible for participation, individuals needed to be 18 years of age or older; be HIV‐positive; not require urgent medical attention; have internet access and have a valid email address; and also read and accept the informed consent. Exclusion criteria were set to ensure that participants could safely complete the procedures and to minimize confounding interpretation of our findings, which encompassed factors such as pharmacological treatments (e.g. anxiolytics and antidepressants; past 6 weeks); concurrent research, psychotherapy, or empirically supported treatments for depression; and history of major neurological disorder or moderate to severe traumatic brain injury or psychotic disorders. Data were collected online over two time points, at baseline (Time 1 [T1]) and 2 weeks post‐intervention (Time 2 [T2]).^3^

**FIGURE 1:**
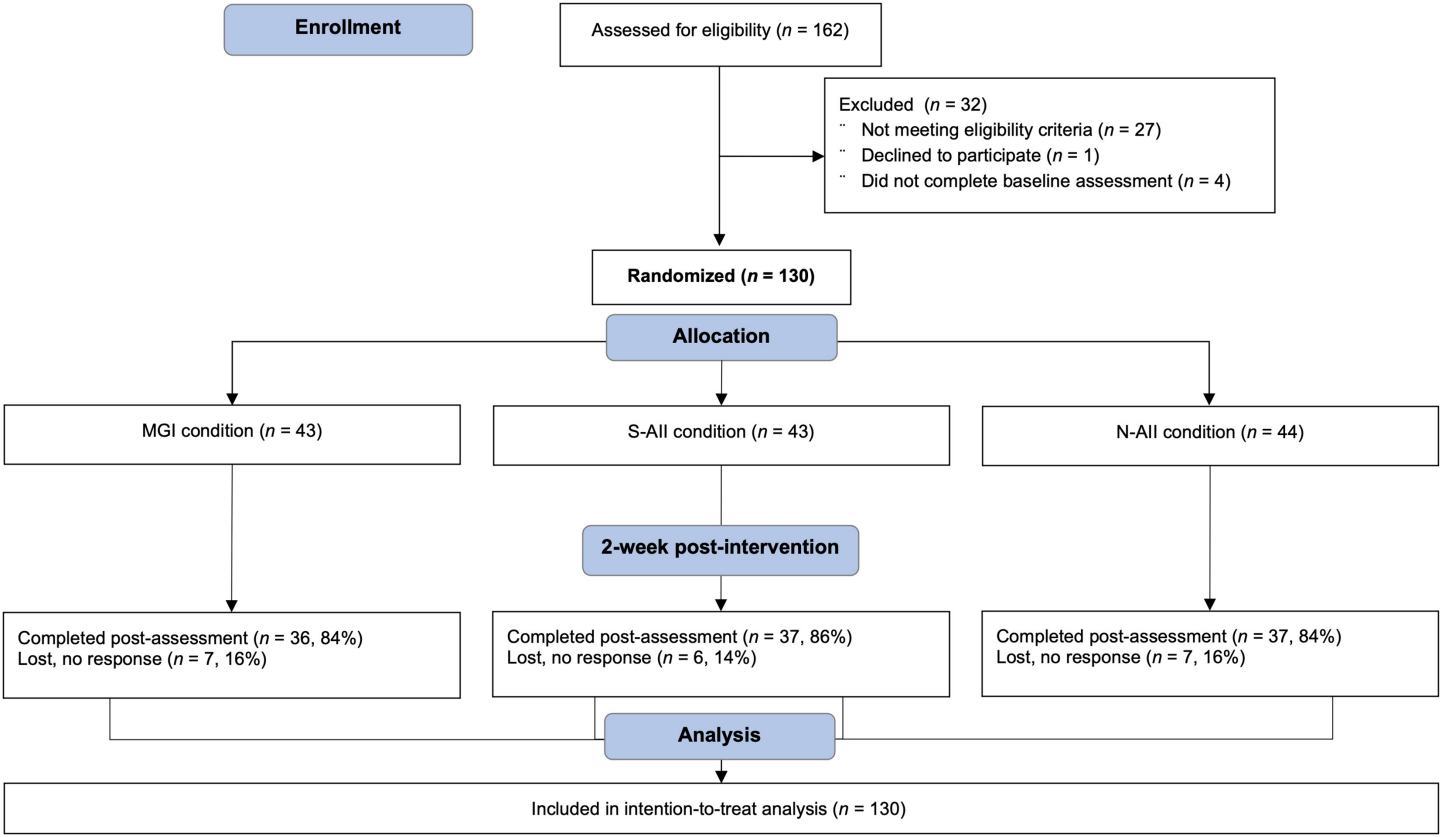
CONSORT flow diagram: Enrollment, group randomization, attrition, and data analysis. MGI, mere goal intention condition; N‐AII, non‐affirming implementation intention condition; S‐AII, self‐affirming implementation intention condition

To secure adequate power, a minimum sample size of 120 participants (40 per group) was estimated, using G*Power v3.1 (Faul et al., 2007), with the assumption of detecting at least medium effect (*f* = .25) of S‐AII (cf. Morgan & Atkin, 2016; Morgan & Harris, 2015; see also Gollwitzer & Sheeran, 2006; Webb et al., 2012) in a mixed‐model analysis of variance with three groups and two points of measurement, with power set to 80 percent, a significance level of 5 percent, and a correlation of .50 between measures. Anticipating a 20 percent dropout rate, we attempted to recruit 144 participants. Participant recruitment, randomization, and progress through the study are presented in the CONSORT flowchart in Figure 1.

The study information sheet informed participants that they would be asked to complete a set of questionnaires on mood, health‐related outcomes, and disease management, along with a written task concerning their activities for upcoming weeks, without specifying conditions; thus, participants were unaware of the group assignments. After baseline assessment, participants were randomized to one of the three study conditions: (i) S‐AII condition, (ii) N‐AII condition (active control), or (iii) MGI condition (passive control). As randomization to groups was conducted automatically, the research team was blinded to allocation too. A system automatically provided each individual with a unique identification code.

A total of 162 adults were screened for eligibility, of whom 130 completed the baseline assessment and were randomized to S‐AII, N‐AII, or MGI through the Qualtrics randomizer feature. Of the individuals in the randomized sample, the majority were male (90.8%), single (57.7%), with high education levels (59.2%), and paid employment (83.1%). Participants were aged 18 to 74 years (*M* = 39.45 years, *SD* = 12.29) and reported on average good self‐perceived general health (World Health Organization [WHO], 2002), of *M* = 4.09 (*SD* = 0.78). For a detailed characteristic of randomized groups, see Table 1.

**TABLE 1:** Baseline characteristics of participants in the trial (*N* = 130)

	MGI (*n* = 43)	S‐AII (*n* = 43)	N‐AII (*n* = 44)	Test statistics
Demographics				
Age (years), *M* (*SD*)	41.07 (13.34)	37.02 (11.83)	40.23 (11.56)	*F*(2, 127) = 1.31, *p* = .275
Gender, *n* (%)				*χ* ^2^(2, *N* = 130) = 0.02, *p* = .999
Female	4 (9.3%)	4 (9.3%)	4 (9.1%)	
Male	39 (90.7%)	39 (90.7%)	40 (90.9%)	
Marital status, *n* (%)				*χ* ^2^(2, *N* = 130) = 0.21, *p* = .901
Married/cohabiting	15 (34.9%)	17 (39.5%)	16 (36.4%)	
Not married (single, divorced, widowed)	28 (65.1%)	26 (60.5%)	28 (63.6%)	
Education level (highest level completed), *n* (%)				*χ* ^2^(4, *N* = 130) = 5.19, *p* = .269
Low (primary school, lower secondary)	0 (0.0%)	2 (4.7%)	1 (2.3%)	
Intermediate (upper secondary education)	18 (41.9%)	17 (39.5%)	12 (27.3%)	
High (tertiary education, university degree)	25 (58.1%)	24 (55.8%)	31 (70.4%)	
Work status, *n* (%)				*χ* ^2^(6, *N* = 130) = 4.85, *p* = .563
Student	1 (2.3%)	3 (7.0%)	2 (4.6%)	
Paid employment	35 (81.4%)	37 (86.0%)	36 (81.8%)	
Unemployed	1 (2.3%)	1 (2.3%)	3 (6.8%)	
Pensioner or retired	6 (14.0%)	2 (4.7%)	3 (6.8%)	
Health‐related characteristics				
Present general health, *M* (*SD*)	4.07 (0.83)	4.14 (0.74)	4.05 (0.78)	*F*(2, 127) = 0.17, *p* = .845
CD4^+^ cell counts, *n* (%)				*χ* ^2^(4, *N* = 130) = 3.47, *p* = .483
<200 cells/μl	6 (13.9%)	9 (20.9%)	4 (9.1%)	
200–500 cells/μl	11 (25.6%)	13 (30.2%)	16 (36.4%)	
>500 cells/μl	26 (60.5%)	21 (48.9%)	24 (54.5%)	
HIV‐1 viremia, *n* (%)				*χ* ^2^(4, *N* = 130) = 6.58, *p* = .160
Undetectable viral load	38 (88.3%)	36 (83.7%)	42 (95.5%)	
40–100,000 copies/ml	2 (4.7%)	5 (11.6%)	2 (4.5%)	
>100,000 copies/ml	3 (7.0%)	2 (4.7%)	0 (0.0%)	
Antiretroviral treatment duration in years, *M* (*SD*)	7.15 (6.93)	6.16 (5.51)	6.61 (6.23)	*F*(2, 127) = 0.27, *p* = .764
Primary outcomes				
PHQ‐9, *M* (*SD*)	4.49 (4.42)	5.44 (4.45)	5.66 (5.06)	*F*(2, 127) = 0.80, *p* = .454
Prevalence of depression, *n* (%)				*χ* ^2^(4, *N* = 130) = 6.11, *p* = .191
No symptoms (PHQ‐9 ≤ 4)	25 (58.2%)	17 (39.5%)	25 (56.8%)	
Mild symptoms (PHQ‐9 ≥ 5 and ≤9)	13 (30.2%)	17 (39.5%)	9 (20.5%)	
Significant symptoms (PHQ‐9 ≥ 10)	5 (11.6%)	9 (21.0%)	10 (22.7%)	
MHC‐SF (total score), *M* (*SD*)	38.49 (15.18)	36.98 (15.92)	37.55 (16.14)	*F*(2, 127) = 0.10, *p* = .904
MHC‐SF EW, *M* (*SD*)	7.81 (3.72)	8.77 (4.39)	8.91 (4.10)	*F*(2, 127) = 0.92, *p* = .400
MHC‐SF SW, *M* (*SD*)	10.79 (6.38)	9.35 (5.70)	9.93 (6.76)	*F*(2, 127) = 0.57, *p* = .567
MHC‐SF PW, *M* (*SD*)	19.88 (6.59)	18.86 (7.23)	18.70 (7.07)	*F*(2, 127) = 0.37, *p* = .695
Secondary outcomes				
Positive self‐directed feelings, *M* (*SD*)	17.86 (4.02)	16.33 (4.36)	16.32 (4.46)	*F*(2, 127) = 1.86, *p* = .161
Positive other‐directed feelings, *M* (*SD*)	18.35 (3.62)	17.67 (3.99)	17.23 (4.73)	*F*(2, 127) = 0.81, *p* = .449

Abbreviations: EW, emotional well‐being subscale; MGI, mere goal intention condition; MHC‐SF, Mental Health Continuum—Short Form; N‐AII, non‐affirming implementation intention condition; PHQ‐9, nine‐item Patient Health Questionnaire; PW, psychological well‐being subscale; S‐AII, self‐affirming implementation intention condition; SW, social well‐being subscale.

### Intervention materials

#### Self‐affirming implementation intention condition (S‐AII)

The S‐AII is a brief means of affirming self in which participants are asked to form an implementation intention with self‐affirmation (cf. Armitage et al., 2011; see also Morgan & Atkin, 2016; Morgan & Harris, 2015). Basically, each individual is asked to formulate an if–then plan with self‐affirming cognitions, for example: “If I feel sad, threatened, or uneased by something, then I will think about the things I value about myself.” Participants are provided with an implementation intention prompt in the form of a sentence stem: “If I feel sad, threatened or uneased by something, then I will …,” where “feeling sad, threatened, or uneased” is the anticipated critical situation. A choice of self‐affirming responses with which to complete the sentence includes, e.g. “… think about my values”; “… think about the things I value about myself”; “… remember things that I have succeeded at”; and “… think about the people who are important to me” (cf. Armitage et al., 2011; Harris et al., 2019). Participants were provided with six options representing a focus on personally important values, strengths/attributes, and social relationships—two per each of the three domains of self‐affirmation. They were asked to point to the preferred response using a checkbox and type out both the stem and their chosen option on given blank lines. Finally, they were asked to read the plan three times, repeat it silently to themselves, and memorize it.

#### Non‐affirming implementation intention condition (N‐AII)

Participants in the active control group received the instruction to formulate an if–then plan with distraction strategies (cf. Morgan & Atkin, 2016; Morgan & Harris, 2015). This active control condition was chosen as the comparator to provide greater control equivalence in self‐affirmation intervention research (see Napper et al., 2009) and to allow the effect of the S‐AII intervention to be examined above and beyond the effect of setting non‐affirming implementation intentions. Individuals were provided with an implementation intention prompt in the form of a sentence stem: “If I feel sad, threatened or uneased by something, then I will ….”. This was followed by six options, with no opportunity to self‐affirm, for example: “… think about the shops and buildings I pass on a journey I travel regularly”; “… think about the best flavor for ice‐cream”; “… remember the food I have eaten in the last 48 hours”; and “… think about the most satisfying season of the year” (cf. Morgan & Atkin, 2016; Morgan & Harris, 2015). Thus, participants in the N‐AII condition were given the same sentence stem as those in the S‐AII condition; however, the six options provided were based on distraction strategies to ensure that there is no opportunity for participants to self‐affirm. Participants completed the task by ticking a box with one preferred distraction strategy and typing out the full plan. Finally, they were instructed to read the plan three times, repeat it silently to themselves, and memorize it.

#### Mere goal intention condition (MGI)

This passive control condition was chosen as the basic comparator to allow the effect of the intervention to be examined above and beyond the effect of simply setting a goal intention. Participants in this control group received instruction to identify and form a goal intention regarding adaptive functioning and feeling good in the next few weeks (i.e. an intention in the format “I want to achieve outcome X/perform behavior X!”, with X representing desired future, outcome, or behavior) (cf. Gollwitzer, 2014). They were asked to type out the goal intention they set. Finally, participants were instructed to read their goal three times, repeat it silently to themselves, and memorize it.

### Measures

At baseline, participants completed an online assessment of demographic factors and health‐related outcomes (i.e. self‐perceived general health, diagnosis of AIDS‐defining clinical conditions, CD4 cell counts, and a level of HIV‐1 viremia in the last laboratory test). Present general health (WHO, 2002) was measured by asking participants: “In general, how would you rate your health today?”, with the possible choices being from 1 (*very bad*) to 5 (*very good*). CD4 count was assessed by asking participants to indicate the level of CD4 cells in their last laboratory test, with the possible choices being “below 200 cells/μl,” “200–500 cells/μl,” and “above 500 cells/μl”. A level of HIV‐1 viremia was assessed by asking participants to indicate the rate of viremia in their last laboratory test, with the possible choices being “viral load of an undetectable level,” “viral load of 40–100,000 copies/ml,” and “viral load above 100,000 copies/ml”. Participants were also asked whether they have been diagnosed with one or more listed AIDS‐defining clinical conditions (cf. Schneider et al., 2008). Primary and secondary outcomes were assessed at baseline and 2 weeks post‐intervention.

#### Primary outcomes

The nine‐item Patient Health Questionnaire (PHQ‐9; Kroenke et al., 2001) was used to measure depression severity. Items are rated on a 4‐point Likert‐type scale from 0 (*not at all*) to 3 (*nearly every day*). It is one of the most frequently used diagnostic self‐report scales for screening, diagnosis, and severity assessment of major depression (cf. Levis et al., 2019). The PHQ‐9 is well validated against standard criteria. It has demonstrated sensitivity to change and is used in a variety of clinical and nonclinical settings, including HIV‐infected patients (e.g. Blenkiron & Goldsmith, 2019; Horton & Perry, 2016; McMillan et al., 2010). In the current research, Cronbach's alpha coefficient at the first and second assessment points was .87 and .91, respectively.

The 14‐item Mental Health Continuum—Short Form (MHC‐SF; Karaś et al., 2014; Żemojtel‐Piotrowska et al., 2018) was used to measure well‐being. Items are rated on a 6‐point scale ranging from 0 (*never*) to 5 (*every day*). The MHC‐SF assesses three dimensions of well‐being: (i) hedonic, emotional well‐being (three items), which relates to positive emotions and life satisfaction; (ii) eudaimonic, social well‐being (five items), which relates to one's functioning in society (i.e. social contribution, social integration, social growth, social acceptance, and social coherence); and (iii) eudaimonic, psychological well‐being (six items), which relates to optimal individual functioning (i.e. self‐acceptance, environmental mastery, positive relations with others, personal growth, autonomy, and purpose in life). The MHC‐SF is well validated, has demonstrated sensitivity to change, and is used in a variety of clinical and nonclinical settings (e.g. Ferentinos et al., 2019; Weiss et al., 2016). In this study, the total scale scores (overall well‐being), as well as the subscale scores, were used. Cronbach's alpha coefficients for total score and subscale scores through both assessment points ranged from .85 to .96 (*M*_α_ = .92).

#### Secondary outcomes

Positive other‐ and self‐directed feelings were measured by asking participants to indicate how often they have experienced five prosocial (e.g. love, empathic, connected, and grateful) and five positive feelings directed toward themselves (e.g. pride, feeling strong, and in control) in their daily lives, respectively (Crocker et al., 2008; Thomaes et al., 2012). Items were rated on a 5‐point scale, ranging from 0 (*very rarely or never*) to 4 (*very often or always*). In this research, Cronbach's alpha coefficients through assessment points for these scales ranged from .83 to .89 (*M*_α_ = .87).

### Statistical analysis

Primary analyses were conducted using an intention‐to‐treat (ITT) approach, with the inclusion of all randomized participants. To carry through a full intention‐to‐treat analysis, linear mixed models (LMMs) were utilized for primary and secondary outcomes. LMM has excellent characteristics concerning account for natural correlation between repeated measurements, handling missing values, and the use of all available data (Sullivan et al., 2018). All models were fitted with maximum likelihood estimation and an unstructured covariance matrix (cf. Lu & Mehrotra, 2010). Each model tested included a random factor for subjects to account for correlation among repeated measures. Besides, in all models, time, intervention condition, and their interaction were included as fixed factors. Covariates were age and gender. Gender as a covariate was included because the resulting sample was not gender‐balanced and also to regard to the gender differences in mental health problems that have been reported in PLWHA and differential responses to psychological interventions as well (see, e.g. Piccinelli & Wilkinson, 2000; Rezaei et al., 2019; Sherr et al., 2011). Age as a covariate was included because younger age has been shown to be an important risk factor for mental health issues in PLWHA (see, e.g. Feuillet et al., 2017). The ITT analyses were complemented by per‐protocol (PP) analyses in which only the sample with complete cases is included. However, we only report ITT findings, unless divergent results in PP analyses emerged.

Additionally, we report intervention effects using one of the recommended methods to classify participants' change in terms of rates of individuals demonstrating a minimal clinically important difference in main outcomes (MCID; Mouelhi et al., 2020). The MCID is the smallest change in a treatment outcome that can be considered to be worthwhile/clinically important, that is, the level of change an individual would identify as important and which would indicate a change in the patient's management (Mouelhi et al., 2020; Wright et al., 2012). MCID is a context‐specific value. To estimate the intraindividual MCID, a standard error of measurement (SEM) is used (cf. Wright et al., 2012; Wyrwich et al., 1999). Using a 2‐SEM change criterion (a more conservative estimate) is considered a suitable method for evaluating clinically relevant change in individual patient scores (Kroenke et al., 2016; Löwe et al., 2004; Wright et al., 2012). In the current study, to establish intraindividual MCIDs that reflect the 95 percent confidence interval, the estimated SEMs for the PHQ‐9 and MHC‐SF were multiplied by 1.96, aligning with the more conservative approach. The MCID values for the PHQ‐9 and MHC‐SF were established to 4 and 7 scores, respectively. Based on these criteria, a clinically meaningful improvement or a clinically meaningful deterioration in the primary outcomes was determined.

## RESULTS

### Randomization check and attrition analysis

A series of ANOVA and chi‐squared tests indicated that the study conditions did not significantly differ regarding sociodemographic characteristics (i.e. age, gender, education, employment, or marital status), health‐related variables (including CD4 cell count, HIV viral load levels, and years of taking antiretroviral treatment), and, most importantly, primary and secondary variables at baseline (all *p*s ≥ .160), indicating successful randomization (see Table 1).

Retention rates at T2 were 86 percent (*n* = 37), 84 percent (*n* = 37), and 84 percent (*n* = 36) for S‐AII, N‐AII, and MGI conditions, respectively. Attrition between randomization and completion of the post‐intervention assessment was found to not differ by condition. Also, no significant differences were found between dropouts and completers on key study variables, all *p*s > .304. These results indicate that the dropout was non‐systematic.

### Intervention effects on primary outcomes

Table 2 demonstrates an overview of the results of LMMs on depressive symptoms and overall well‐being, controlling for age and gender specified as covariates. Estimated means are presented in the supporting information. Analyses revealed no significant main or interaction effects for depression, but a significant time × condition interaction for well‐being. For exploratory purposes, analyses were also conducted for three dimensions of well‐being. Results showed significant interaction effects for emotional and social dimensions of well‐being, and a marginal interaction effect for psychological well‐being (see Table 3).

**TABLE 2:** Results of the LMM analysis for primary and secondary outcomes

	Overall well‐being				Depression				Positive self‐directed feelings				Positive other‐directed feelings			
	Estimate (*SE*)	*df*	*t*	*p*	Estimate (*SE*)	*df*	*t*	*p*	Estimate (*SE*)	*df*	*t*	*p*	Estimate (*SE*)	*df*	*t*	*p*
Intercept	14.13 (10.73)	130	1.32	.190	11.32 (3.20)	130	3.54	<.001	13.03 (2.85)	128	4.57	<.001	17.95 (2.82)	126	6.37	<.001
Gender	5.43 (4.61)	128	1.18	.241	−2.10 (1.37)	128	−1.53	.127	0.78 (1.22)	127	0.65	.518	−1.29 (1.21)	124	−1.07	.287
Age	0.33 (0.11)	132	3.01	.003	−0.05 (0.03)	133	−1.61	.110	0.06 (0.03)	132	1.95	.053	0.06 (0.03)	131	1.89	.061
Time	0.01 (0.78)	113	0.02	.986	−0.25 (0.30)	115	−0.85	.396	−0.02 (0.26)	113	−0.06	.952	−0.18 (0.28)	111	−0.62	.534
Condition: MGI vs. SA‐II, NA‐II	0.42 (2.88)	147	0.14	.885	−0.93 (0.88)	158	−1.07	.288	0.87 (0.75)	130	1.16	.247	0.43 (0.74)	128	0.58	.562
Condition: SA‐II vs. NA‐II	0.51 (3.32)	147	0.16	.877	−0.40 (1.01)	158	−0.39	.698	0.56 (0.86)	130	0.66	.512	0.73 (0.85)	128	0.86	.390
Time × condition: MGI vs. SA‐II, NA‐II	−2.60 (1.66)	113	−1.57	.120	0.10 (0.63)	115	0.17	.868	−1.06 (0.56)	113	−1.89	.061	−0.67 (0.60)	111	−1.11	.268
Time × condition: SA‐II vs. NA‐II	4.53 (1.90)	113	2.39	.019	−0.35 (0.72)	115	−0.48	.630	0.74 (0.65)	113	1.15	.255	0.22 (0.69)	111	0.32	.749

Abbreviations: *df*, degrees of freedom (Satterthwaite method for degrees of freedom); LMM, linear mixed model; MGI, mere goal intention condition; N‐AII, non‐affirming implementation intention condition; S‐AII, self‐affirming implementation intention condition.

**TABLE 3:** Results of the LMM analysis for three dimensions of well‐being

	Psychological well‐being				Emotional well‐being				Social well‐being			
	Estimate (*SE*)	*df*	*t*	*p*	Estimate (*SE*)	*df*	*t*	*p*	Estimate (*SE*)	*df*	*t*	*p*
Intercept	7.61 (4.73)	129	1.61	.110	2.16 (2.64)	130	0.82	.415	4.35 (4.28)	129	1.02	.311
Gender	3.06 (2.03)	128	1.51	.134	1.22 (1.14)	128	1.08	.285	1.15 (1.84)	128	0.63	.533
Age	0.14 (0.05)	133	2.83	.005	0.10 (0.03)	134	3.80	<.001	0.09 (0.04)	132	2.07	.040
Time	−0.44 (0.39)	114	−1.11	.268	0.18 (0.24)	115	0.76	.451	0.28 (0.35)	113	0.79	.430
Condition: MGI vs. SA‐II, NA‐II	−0.07 (1.24)	131	−0.06	.957	−1.12 (0.69)	132	−1.62	.108	0.30 (1.12)	131	0.27	.788
Condition: SA‐II vs. NA‐II	1.08 (1.42)	131	0.76	.449	0.97 (0.80)	132	1.22	.223	0.71 (1.29)	131	0.55	.583
Time × condition: MGI vs. SA‐II, NA‐II	−1.67 (0.84)	114	−1.99	.050	0.32 (0.50)	115	0.64	.526	−1.25 (0.75)	113	−1.67	.097
Time × condition: SA‐II vs. NA‐II	0.95 (9.96)	114	0.99	.326	1.56 (0.58)	115	2.71	.008	1.99 (0.86)	113	2.32	.022

Abbreviations: *df*, degrees of freedom (Satterthwaite method for degrees of freedom); LMM, linear mixed model; MGI, mere goal intention condition; N‐AII, non‐affirming implementation intention condition; S‐AII, self‐affirming implementation intention condition.

Regarding overall well‐being, post hoc analyses showed significant change over time, but only within the S‐AII condition (*M*_diff_ = 3.14, 95% CI 0.48–5.80; *p* = .021; *d* = .23). In terms of the emotional dimension of well‐being, significant group differences with medium effect sizes were observed between the S‐AII and MGI conditions (*M*_diff_ = 1.83, *SE* = 0.88, *p* = .039; *d* = .45) and S‐AII and N‐AII conditions (*M*_diff_ = 1.75, *p* = .047; *d* = .43). Moreover, based on the within‐group estimates, only within the S‐AII a significant change was detected (*M*_diff_ = 0.85, *SE* = 0.88, *p* = .039; *d* = .24). Similarly, post hoc analyses revealed also a significant change over time on the social dimension of well‐being, but only within the S‐AII condition (*M*_diff_ = 1.69, 95% CI 0.49–2.89; *p* = .006; *d* = .30). Finally, in terms of the psychological dimension of well‐being, only within the control group—MGI condition—a significant change was observed, suggesting deterioration (*M*_diff_ = −1.55, *SE* = 0.70, *p* = .028; *d* = −.24). No other significant differences were detected.

In line with the results presented above, MCID indexed significantly higher rates of reliable and clinically important positive changes on well‐being in the S‐AII group relative to both active and passive comparison conditions (see Table 4).

**TABLE 4:** MCID proportions in well‐being and depressive symptoms across the three study conditions

	MGI (*n* = 36)	S‐AII (*n* = 37)	N‐AII (*n* = 37)	Test statistics
MCID on the MHC‐SF scores				*χ* ^2^(4, *N* = 110) = 13.29, *p* = .010
Improvement, *n* (%)	6 (16.7%)	15 (40.5%)	4 (10.8%)	
No change, *n* (%)	18 (50.0%)	18 (48.7%)	24 (64.9%)	
Deterioration, *n* (%)	12 (33.3%)	4 (10.8%)	9 (24.3%)	
MCID on the PHQ‐9 scores				*χ* ^2^(4, *N* = 110) = 5.83, *p* = .212
Improvement, *n* (%)	3 (8.3%)	9 (24.3%)	5 (13.5%)	
No change, *n* (%)	31 (86.1%)	23 (62.2%)	28 (75.7%)	
Deterioration, *n* (%)	2 (5.6%)	5 (13.5%)	4 (10.8%)	

*Note*. Results are based on complete case analysis.

Abbreviations: MCID, minimal clinically important difference; MGI, mere goal intention condition; MHC‐SF, Mental Health Continuum—Short Form; N‐AII, non‐affirming implementation intention condition; PHQ‐9, nine‐item Patient Health Questionnaire; S‐AII, self‐affirming implementation intention condition.

### Intervention effects on secondary outcomes

LMM analyses for positive self‐ and other‐directed feelings, controlling for age and gender, revealed no significant effects of time, condition, or their interactions (see Table 2).

## DISCUSSION

This trial was designed to provide the first evaluation of the effectiveness of a brief psychological intervention, known as self‐affirming implementation intentions, in improving mental health outcomes among PLWHA. The results of this study indicate positive short‐term effects of the intervention (i.e. over 2 weeks) on overall well‐being, but not in terms of depressive symptoms. All issues of particular importance for a better understanding of the obtained pattern of findings are discussed, emphasizing issues that may direct cumulative progress and further refinements of self‐affirmation interventions in further research.

In contrast with using laboratory‐induced stressors, this study directly compares the effects of self‐affirmation on the generation of both positive effects—a net benefit on psychological functioning (i.e. boosting well‐being)—and the reduction of negative effects (i.e. decreasing depressive symptoms) among those at risk of mental health problems, experiencing chronic/acute stressors. Although findings from this study failed to confirm the effectiveness of S‐AII in reducing depressive symptoms in PLWHA, the S‐AII status as an effective well‐being intervention was empirically supported. Our findings are thus partially in line with the recent research on the S‐AII effectiveness in adults with psoriasis that showed both significant improvements in well‐being (*d*s > .25) and reductions of depressive (*d*s > −.40) and anxiety symptoms (*d*s > −.45) (Łakuta, 2021). In the present study, the S‐AII intervention yielded small to medium effects on overall well‐being and its emotional and social dimensions (*d*s from .23 to .45). To shed more light on these results, the mean estimated effect sizes of positive psychology interventions are reported to be .34 on subjective well‐being, .20 on psychological well‐being, and .23 on depression (Bolier et al., 2013), or, as it has recently been reported, even smaller effects are observed (see White et al., 2019). Here, as noted, though significant improvements in well‐being were observed, there were null effects on depressive symptoms.

The most potent premise for the explanation of the null results in the current study for depressive symptoms is a floor effect. Simply, most of the participants, which are more than 80 percent, reported no or only mild depressive symptoms (PHQ‐9 score ≤9) at baseline. As noted by Sin and Lyubomirsky (2009), in clinical samples, such positive psychological interventions are more likely to show robust effects, yielding medium–large effect sizes; in nonclinical samples, such effects may be much harder to be observed. Thus, further research on S‐AII should be encouraged, albeit applying more restricted exclusion criteria. Nonetheless, this issue has two faces. Exclusionary practices are tempting, but they result in a risk of eliminating a large proportion of a representative cohort of individuals from trial participation, limiting the generalizability of the findings, reducing the confidence that findings can be translated into real‐world settings, and, finally, there is a high risk of reporting overestimated effects.

There is also another issue that can shed more light on these findings. This study embraced only the short‐term evaluation of the intervention effects (i.e. over 2 weeks). Evaluating depressive symptoms for a longer time period, applying a longitudinal design, could provide a better evaluation of the effectiveness of the intervention and a more precise estimate of possible differences across conditions. It has been shown that self‐affirmation interventions with small or subtle initial benefits can set in motion a process whose full consequences accumulate over time (Goyer et al., 2017). Regarding the observed positive effects on social and emotional dimensions of well‐being, the S‐AII effects seem promising, and future research should seek to systematically evaluate trajectories in both positive and negative mental health outcomes. It is also notable that at the same time, within the control group—MGI condition—a significant deterioration in terms of the psychological dimension of well‐being was observed (*d* = −.24), with no such effect in the S‐AII group. Thus, a replication of the current findings with a longer term evaluation is warranted. Otherwise, interventions whose benefits are slowly to fully develop, but their effects may last longer, could be ousted unfairly.

Finally, the considerable improvements in well‐being in 40.5 percent of participants as the result of the S‐AII intervention are very encouraging. Nonetheless, it is also important to note that based on MCID indexes, the S‐AII intervention resulted in a deterioration in approximately 10–14 percent of participants. These findings suggest a low level of person‐activity fit for some individuals (Layous & Lyubomirsky, 2014; see also Howell, 2017) and/or that some important factors (e.g. personal, contextual, and/or processual) might not be well captured within the S‐AII intervention, which results in limited effectiveness for some groups of people. As suggested by Ferrer and Cohen (2019), providing direct resources to support active change may enhance self‐affirmation benefits (e.g. via behavioral activation to guide positive activities). As such, further systematic research is needed on the optimization of self‐affirmation interventions, before their application in real‐life contexts.

### Strengths, limitations, and future research

These findings are drawn from the preregistered RCT with sufficient^4^ (assumed) statistical power. Notably, the study employed passive and active comparison groups, matched to the target condition, providing a more robust evaluation.^5^ Another strength of this study is the low attrition rate. Furthermore, the adopted statistical approach (i.e. LMM) enabled us to maximize the use of data from each participant in parameter estimation and significance testing, producing more reliable estimates (cf. Sullivan et al., 2018). Moreover, the adopted means of self‐affirmation in this study, that is, the S‐AII, though different by design from typical self‐affirmation writing exercises (see McQueen & Klein, 2006), was successfully tested in prior research in terms of its essential efficacy and effectiveness (Armitage et al., 2011; see also Armitage, 2016; Łakuta, 2020, 2021; Morgan & Atkin, 2016; Morgan & Harris, 2015); thus, it cannot per se explain the null results in the present study.

There are however several limitations in this trial that should be considered thoroughly. Participants in this study represented only a segment of the community population that was interested in actively responding to the study. Moreover, the sample was not gender‐balanced, and women were underrepresented,^6^ unfortunately, being a typical issue in research among PLWHA (see, e.g. Sherr et al., 2011). The limitations in terms of our sample provide us with a good understanding of the group of men living with HIV, but it is difficult to know to what extent these findings can be generalized more broadly to women, and also older individuals, or adolescents. Additionally, more strict inclusion criteria to be included in the trial were not adopted (e.g. having at least a moderate level of depressive symptoms). Another limitation concerns using solely self‐reported measures. Although the tools used are well validated, with good sensitivity to change, future investigations are encouraged using also informant reports along with an ecological momentary assessment (EMA) to improve the evaluation of intervention effectiveness. Of note, the EMA approach offers more sensitivity in detecting changes; moreover, its higher precision in measuring intervention effectiveness allows determining how intervention effects vary over time (Moore et al., 2016; Schuster et al., 2020).

## CONCLUSIONS

The current study provides evidence that supports the S‐AII status as an effective intervention in improving well‐being in PLWHA. Specifically, the intervention yielded primarily positive effects on social and emotional dimensions of well‐being. These findings are notable given that the intervention was brief and low in intensity and its effects were not augmented by the inclusion of any booster component. The S‐AII effects were compared with both the passive and active comparison groups, providing more robust evidence for the utility and the effectiveness of the intervention. We hope that these first RCT findings will catalyze further research on self‐help interventions for PLWHA, with long‐term follow‐up.

## CONFLICT OF INTERESTS

None.

## ETHICS STATEMENT

The study was performed with ethical approval granted by the University's Institutional Review Board and in accordance with the ethical standards as laid down in the 1964 Declaration of Helsinki and its later amendments or comparable ethical standards.

## CONSENT TO PARTICIPATE

Informed consent was obtained from all individual participants included in the study.

## Supporting information


**Table S1.** Estimated marginal means for primary and secondary outcomes across all study groups based on LMMs.
**Table S2.** Estimated marginal means for three dimensions of well‐being across all study groups based on LMMs.

## Data Availability

The data that support the findings of this study are available on request from the corresponding author. The data are not publicly available due to privacy and/or ethical restrictions.

## ENDNOTES

1 The full study protocol can be accessed at https://osf.io/z9amq.

2 Qualtrics research platform was used to create a web‐based eligibility screening assessment. To efficiently conduct the recruitment process, a URL or QR code linking to a web page with the survey was supplied to potential participants.

3 At T2, participants received an email with access to measures via the Qualtrics platform. Participants who missed a measurement session received reminder emails (up to three triggers via the system). Data were matched based on participants' unique identification codes (a system automatically provided each individual with a unique identification code at T1).

4 However, it should be kept in mind that to estimate smaller differences in the intervention effects than assumed in a priori power analysis, a larger sample size would be needed.

5 Note that the control group was matched to allow the effect of the S‐AII to be examined above and beyond the effect of simply setting a goal intention, so any conclusions of this study should be seen relative to the efficacy of that control condition (rather than relative to no treatment).

6 However, given that in past self‐affirmation studies most samples were predominantly female, the current findings derived mostly from the male sample represent an important contribution to the existing self‐affirmation literature.
