# Supplementary material for: Enhancing well‐being and alleviating depressive symptoms in people with HIV/AIDS: An intervention based on if–then plans with self‐affirming cognitions
Source: Appl Psychol Health Well Being. 2022 Mar 16;14(3):899–919. doi: 10.1111/aphw.12357 (PMC9540674; doi:10.1111/aphw.12357)
Supplement: Supplementary file 1 — Table S1. Estimated marginal means for primary and secondary outcomes across all study groups based on LMMs. Table S2. Estimated marginal means for three dimensions of well‐being across all study groups based on LMMs. [file APHW-14-899-s001.docx]

Supplementary Material

**Enhancing well‐being and alleviating depressive symptoms in people with HIV/AIDS: An intervention based on if–then plans with self‐affirming cognitions**

**Table S1.** Estimated marginal means for primary and secondary outcomes across all study groups based on LMMs.

|  | | Depression | | | Well-being | | | Positive self-directed feelings | | | Positive other-directed feelings | | |
| --- | --- | --- | --- | --- | --- | --- | --- | --- | --- | --- | --- | --- | --- |
| Group | Time | *M* | *SE* | 95% CI | *M* | *SE* | 95% CI | *M* | *SE* | 95% CI | *M* | *SE* | 95% CI |
| MGI | 1 | 5.44 | 0.92 | 3.63;  7.26 | 35.7 | 3.04 | 29.6;  41.7 | 17.4 | 0.82 | 15.8;  19.0 | 18.8 | 0.81 | 17.2;  20.4 |
| S-AII | 1 | 6.18 | 0.90 | 4.41;  7.96 | 35.5 | 2.98 | 29.6;  41.4 | 16.1 | 0.80 | 14.5;  17.7 | 18.3 | 0.80 | 16.8;  19.9 |
| N-AII | 1 | 6.57 | 0.91 | 4.78;  8.37 | 35.0 | 3.01 | 29.0;  40.9 | 15.9 | 0.81 | 14.3;  17.5 | 17.7 | 0.80 | 16.1;  19.3 |
| MGI | 2 | 5.26 | 0.93 | 3.42;  7.10 | 33.9 | 3.08 | 27.9;  40.0 | 16.7 | 0.83 | 15.1;  18.4 | 18.2 | 0.83 | 16.5;  19.8 |
| S-AII | 2 | 5.72 | 0.92 | 3.92;  7.53 | 38.6 | 3.01 | 32.7;  44.6 | 16.8 | 0.82 | 15.2;  18.5 | 18.5 | 0.82 | 16.9;  20.1 |
| N-AII | 2 | 6.46 | 0.92 | 4.64;  8.29 | 33.6 | 3.04 | 27.6;  39.6 | 15.9 | 0.82 | 14.3;  17.5 | 17.6 | 0.82 | 16.0;  19.3 |

*Note*. MGI = mere goal intention condition; S-AII = self-affirming implementation intention condition; N-AII = non-affirming implementation intention condition.

**Table S2.** Estimated marginal means for three dimensions of well‐being across all study groups based on LMMs.

|  | | Psychological well-being | | | Emotional well-being | | | Social well-being | | |
| --- | --- | --- | --- | --- | --- | --- | --- | --- | --- | --- |
| Group | Time | *M* | *SE* | 95% CI | *M* | *SE* | 95% CI | *M* | *SE* | 95% CI |
| MGI | 1 | 18.4 | 1.35 | 15.7;  21.0 | 7.12 | 0.76 | 5.63;  8.62 | 10.15 | 1.22 | 7.74;  12.6 |
| S-AII | 1 | 17.9 | 1.32 | 15.3;  20.5 | 8.50 | 0.74 | 7.03;  9.96 | 9.08 | 1.20 | 6.72;  11.4 |
| N-AII | 1 | 17.3 | 1.34 | 14.7;  19.9 | 8.30 | 0.75 | 6.82;  9.78 | 9.37 | 1.21 | 6.98;  11.8 |
| MGI | 2 | 16.8 | 1.37 | 14.1;  19.5 | 7.51 | 0.77 | 6.00;  9.03 | 9.59 | 1.24 | 7.15;  12.0 |
| S-AII | 2 | 18.5 | 1.34 | 15.9;  21.2 | 9.35 | 0.75 | 7.86;  10.84 | 10.77 | 1.21 | 8.37;  13.2 |
| N-AII | 2 | 17.0 | 1.36 | 14.3;  19.6 | 7.60 | 0.76 | 6.09;  9.10 | 9.07 | 1.23 | 6.65;  11.5 |

*Note*. MGI = mere goal intention condition; S-AII = self-affirming implementation intention condition; N-AII = non-affirming implementation intention condition.
